# Biomechanical Characteristics of Osteoporotic Fracture Healing in Ovariectomized Rats: A Systematic Review

**DOI:** 10.1371/journal.pone.0153120

**Published:** 2016-04-07

**Authors:** Lin Chen, Long Yang, Min Yao, Xue-Jun Cui, Chun-Chun Xue, Yong-Jun Wang, Bing Shu

**Affiliations:** 1 Longhua Hospital, Shanghai University of Traditional Chinese Medicine, Shanghai, China; 2 Spine Research Institute, Shanghai University of Traditional Chinese Medicine, Shanghai, China; Louisiana State University, UNITED STATES

## Abstract

Biomechanical tests are widely used in animal studies on osteoporotic fracture healing. However, the biomechanical recovery process is still unknown, leading to difficulty in choosing time points for biomechanical tests and in correctly assessing osteoporotic fracture healing. To determine the biomechanical recovery process during osteoporotic fracture healing, studies on osteoporotic femur fracture healing with biomechanical tests in ovariectomized rat (OVX) models were collected from PUBMED, EMBASE, and Chinese databases. Quadratic curves of fracture healing time and maximum load were fitted with data from the analyzed studies. In the fitted curve for normal fractures, the predicted maximum load was 145.56 N, and the fracture healing time was 88.0 d. In the fitted curve for osteoporotic fractures, the predicted maximum load was 122.30 N, and the fracture healing time was 95.2 d. The maximum load of fractured femurs in OVX rats was also lower than that in sham rats at day 84 post-fracture (D84 PF). The fracture healing time was prolonged and maximum load at D84 PF decreased in OVX rats with closed fractures. The maximum load of Wister rats was higher than that of Sprague-Dawley (SD) rats, but the fracture healing time of SD and Wister rats was similar. Osteoporotic fracture healing was delayed in rats that were < = 12 weeks old when ovariectomized, and at D84 PF, the maximum load of rats < = 12 weeks old at ovariectomy was lower than that of rats >12 weeks old at ovariectomy. There was no significant difference in maximum load at D84 PF between rats with an osteoporosis modeling time <12 weeks and > = 12 weeks. In conclusion, fracture healing was delayed and biomechanical property decreased by osteoporosis. Time points around D95.2 PF should be considered for biomechanical tests of osteoporotic femur fracture healing in OVX rat models. Osteoporotic fracture healing in OVX rats was affected by the fracture type but not by the strain of the rat.

## Introduction

The assessment of bone fracture healing is important for both clinical practice and basic research. Current assessment tools can be divided into four categories: imaging analyses, biomechanical tests, detection of serologic markers and clinical examinations [1]. The success of fracture healing should be defined by the restoration of the biomechanical properties of the fractured bone [2]. Therefore, biomechanical tests of the fractured bone are more important and accurate than other assessment tools.

Biomechanical tests are widely used in animal studies on osteoporotic fracture healing [3–5]. However, due to limitations with its usage, the majority of the studies only choose a single time point to conduct the biomechanical test of the fractured bone. Different time points have been adopted in some studies of osteoporotic fracture healing, but the time points were not unified and covered a large range [5–7]. In most cases, the time points were selected according to the pathological changes of the fractured bone. However, as we know, the biomechanical healing process of fractured bone is not the same as the pathological healing process. Therefore, to provide a basis for study designs on osteoporotic fracture healing, particularly regarding the timing of biomechanical tests and evaluation of mechanical indexes, it is important to identify the biomechanical characteristics during the process of osteoporotic fracture healing.

During fracture healing, the mechanical stability of the healing bone steadily increases and eventually reaches the level of intact bone. It is also known that osteoporosis can decrease the biomechanical properties of callus during fracture healing [8]. Therefore, the biomechanical characteristics and processes of osteoporotic fracture healing may be similar to but different from normal fracture healing. In addition, it is also important to identify other factors that may affect the biomechanical quality during osteoporotic fracture healing, such as fracture type, rat strain, osteoporosis modeling time and the age of animals at ovariectomy.

Ovariectomy induces a decline in ovarian production of estrogens which results in a rapid loss of trabecular bone mass and increased bone resorption, consequently increasing the risk for fragility fracture. Based on the similarities in pathogenesis and pathological changes between OVX rat model and postmenopausal osteoporosis in women, The OVX rat has been widely used as a clinically relevant model of human postmenopausal bone loss [9].

Therefore, to better apply biomechanical tests, the aim of this review is to perform a systematic review of the biomechanical recovery process during osteoporotic fracture healing with OVX rat models and to investigate the influence of potential factors on osteoporotic fracture healing.

## Materials and Methods

### Systematic review

The Embase, PubMed, VIP, Wan Fang, CNKI (including China Doctor/Master Dissertation Full Text Database and China Proceedings Conference Full Text Database), and Chinese BioMedical (CBM) databases were included and searched from their inception up to July 2015 with the keywords “rat,” “fracture healing” and “osteoporosis or osteoporotic fracture.” The PUBMED database search strategy was (((((rat [Title/Abstract]) OR rats [Title/Abstract])) AND ((((fracture healing [Title/Abstract]) OR bone fracture [Title/Abstract]) OR heal of fracture [Title/Abstract]) OR union of fracture [Title/Abstract])) AND ((((osteoporosis [Title/Abstract]) OR bone loss [Title/Abstract]) OR osteoporotic [Title/Abstract]) OR osteoporotic fracture [Title/Abstract])). Language restrictions were not applied. Two investigators (LC and LY) selected the studies independently by assessing the titles and abstracts of the publications and obtained copies of the publications according to the inclusion criteria. Disagreements were resolved by consensus with a third author (MY).

### Inclusion criteria

The inclusion criteria for the structured review were as follows: (1) in vivo experimental studies of fracture healing with OVX rat models; (2) use of saline or no treatment as the intervention for the OVX rats; (3) use of the mid-shaft femur as the fracture site and intramedullary fixation for fracture fixation; and (4) a maximal load or ultimate load recorded for biomechanical tests.

### Exclusion criteria

The exclusion criteria were set as the following: (1) use of other osteoporosis models but not ovariectomy-induced osteoporosis, including drug-induced osteoporosis, elderly osteoporosis or studies performed with genetically modified animals; (2) use of other bone fractures but not femur fractures, including vertebral fracture, jaw fracture and fibula fracture; (3) in vitro studies; (4) studies that were not experimental controlled trials (reviews, letters and expert opinion publications); and (5) publications without available full text.

### Data Extraction

For each eligible study, two reviewers independently extracted the available and relevant data using a predefined form, including the name of the first author, year of publication, the strain, age and weight of the rats, numbers of rats in the sham and OVX groups, whether the sham group was a saline/no treatment group, osteoporosis modeling time, fracture modeling method, and the time points of biomechanical test (days post-fracture). For outcome measures, maximal loads or ultimate loads were extracted with either raw data or group averages and standard deviation (SD). Data presented only in graphs were estimated using GetData Graph Digitizer version 2.26 (Fedorov. S, 2013, Getdata-graph-digitizer.com, Russia). A third reviewer (MY) resolved any disagreements between the two reviewers. Necessary data that was not mentioned in the studies were acquired by contacting the authors. If data could not be acquired, the study was excluded from further analysis.

### Assessment of the risk of bias in individual studies

Two reviewers (LC and LY) assessed the risk of bias in each included study. STAIR (the initial Stroke Therapy Academic Industry Roundtable) was used to assess the quality of the studies, including the availability of the following factors: (1) sample size calculation; (2) inclusion and exclusion criteria; (3) randomization; (4) allocation concealment; (5) description of animals excluded from analysis; (6) blinded assessment of the outcome; and (7) potential conflicts of interest and funding support [10, 11]. The quality score of each individual study was calculated.

### Data analysis and synthesis

To obtain the quadratic equation between fracture healing time and maximum load, a non-linear regression model was established using a least squares regression method. The quadratic curve of the maximum load and the fracture healing time was also fitted according to the quadratic equation. The maximum load of the curves and the corresponding fracture healing times were calculated. All data passed the normality test, and the level of significance was set at p < 0.05. All statistical analyses and estimated curves were performed with SPSS 18.0. Summary estimators of maximum load were calculated using relative risk or weighted mean difference (WMD) and 95% confidence intervals (CI).

## Results

### Selection of studies

**Fig 1:**
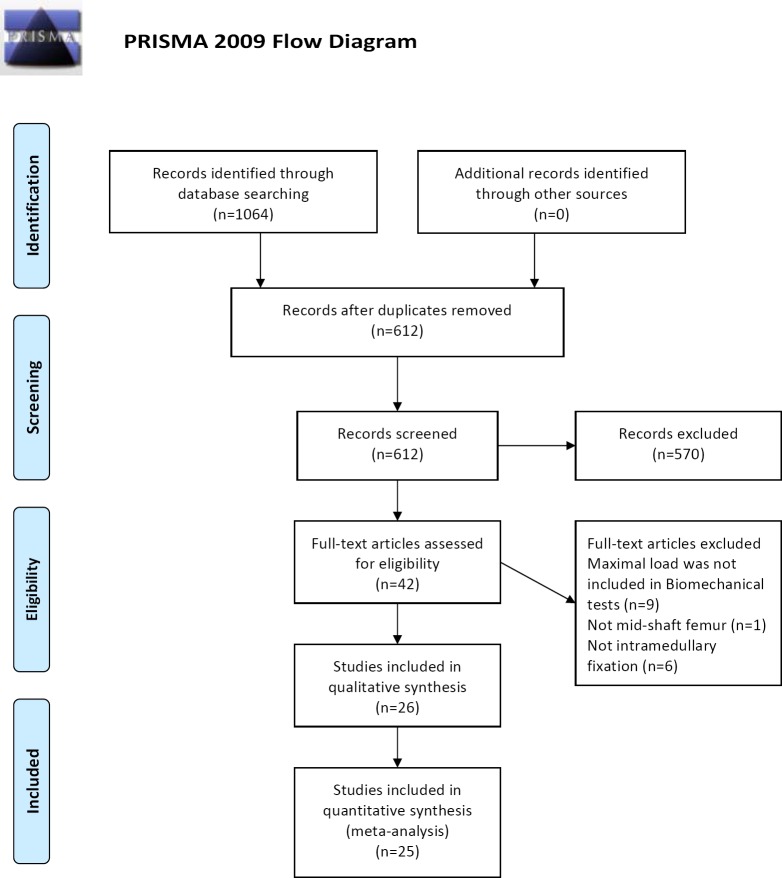
Study flow diagram.

The study selection process is shown in Fig 1. A total of 1064 publications from electronic databases were identified through key word selection. After screening the titles and abstracts, 42 studies were retained. The full text of the 42 remaining studies were read. Of those, 16 studies were excluded because the maximal loads were not included in the biomechanical tests [7, 12–21]; fractures of other bones but not mid-shaft femur fractures were used [22–25]; or a fixation method other than intramedullary fixation was used [26]. Nine of these 16 studies were published in Chinese, and the rest were in English. Finally, 26 studies met the inclusion criteria.

### Characteristics of the included studies

The characteristics of the studies that met the inclusion criteria are shown in Table 1.

**Table 1:** Description of the characteristics of included studies.

Study	Strain of rats	Age of rats at ovariectomy (weeks)	Weight of rats (g)	Number of rats in sham/OVX group	Including sham group or not	Osteoporosis modeling time (weeks)	Fracture modeling method	Time of biomechanical test (days post-fracture)
Hao Y J 2007 [27]	SD	32	N/A	6 / 6	Y	12	Open fracture	84
Wei J S 2014 [28]	Wister	12	245–300	N / 5	N	8	Open fracture	84
Chen L 2013 [29]	SD	24	200–250	N / 6	N	24	Open fracture	56
Namkung-Matthai H 2001 [30]	SD	8	242±2.5	5 / 5	Y	12	Open fracture	21
Kuo Y J 2014 [31]	SD	8	N/A	6 / 6	Y	4	Open fracture	42, 112
Fu L 2009 [6]	SD	24	264±2	N / 6	N	12	Open fracture	42, 112
Zhang C 2009 [32]	SD	32	300–320	N / 6	N	12	Open fracture	42
Zhang G 2014 [33]	SD	16	250–300	N / 5	N	8	Open fracture	28, 84
Xiao D M 2007 [34]	SD	24	260–300	16 / 16	Y	12	Open fracture	63
Li Z Q 2009 [35]	Wister	24	220–280	N / 20	N	12	Open fracture	42
Jin W J 2013 [36]	SD	12	238.4±14	6 / 6	Y	8	Open fracture	56
Huang X H 2012 [37]	Wister	12	260–300	N / 5	N	12	Open fracture	84
Rong J M 2012 [38]	SD	12	240±25	6 / 6	Y	8	Open fracture	42
Zhang C 2008 [39]	SD	32	300–320	N / 3	N	12	Open fracture	42
Dai G C 2013 [40]	SD	12	250±25	8 / 8	Y	8	Open fracture	42
Wang X P 2014 [41]	SD	24	273±16	N / 6	N	12	Open fracture	42, 84
Qiu Z J 2010 [42]	SD	10	220–240	N / 5	N	6	Open fracture	28, 42
Cao G L 2014 [43]	SD	16	270±25	8 / 8	Y	8	Open fracture	56
Sha M 2009 [44]	SD	12	241±16	N / 9	N	12	Open fracture	42, 84
Chow S K 2014 [45]	SD	24	N/A	5 / 5	Y	12	Closed fracture	56
Cheung W H 2012 [46]	SD	24	N/A	6 / 6	Y	12	Closed fracture	28, 56
Mohamad S 2012 [47]	SD	N/A	250–300	8 / 8	Y	8	Closed fracture	56
Blokhuis T J 2012 [48]	Wister	N/A	228±6.1	N/A / N/A	Y	6	Closed fracture	14
Wang L 2005 [49]	SD	18	280–310	N / 6	N	10	Closed fracture	28, 42, 56, 84
Estai M A 2011 [50]	SD	N/A	200–250	10 / 10	Y	6	Closed fracture	42
Shi H F 2010 [51]	SD	24	N/A	8 / 8	Y	12	Closed fracture	56

N/A, not available; Y, yes; N, no

SD rats were used in 22 studies, and Wister rats were used in four studies [22, 35, 37, 48]. The age of the rats when the ovariectomy was performed ranged from 8-weeks to 32-weeks old. Three studies did not include the ages of the rats. The average sample size of the sham group and OVX group of all 26 studies ranged from 3 to 20.

All of the 26 studies used the ovariectomy-induced osteoporosis model, and two of them used an additional low calcium diet [30, 48]. A sham group was created in 14 studies [27, 30, 31, 34, 36, 40, 43, 45–48, 50–52]. One study used two different types of intramedullary nails in each osteoporotic fracture femur [44]. The osteoporosis modeling time ranged from 6 weeks to 24 weeks. An open fracture model was adopted in 19 studies, and closed fracture models were used in the remaining seven studies.

Eighteen studies had only one time point for the biomechanical test, and seven studies had two time points [6, 31, 33, 41, 42, 44, 46]. Only one study had four time points [49]. The length of time from fracture to biomechanical test ranged from 14 days to 112 days.

### Risk of bias within studies

The risk of bias for all 26 studies are shown in Table 2. Seven of the 26 (27%) studies were evaluated as high quality studies [6, 41, 45, 48, 49, 51, 52]. Only one study described the calculation of the sample size [30], and three studies described the inclusion and exclusion criteria [45, 9, 51]. Randomization was reported in 22 studies but not four studies [27, 9, 30, 44], whereas allocation concealment was used in none of the studies. Eleven studies described the animals excluded from analysis. Blinded assessment of the outcome was described in only one study [29]. Fourteen studies reported potential conflicts of interest and funding support. In conclusion, the overall methodological quality of the studies was not high.

**Table 2:** Risk of bias.

Study	Sample size calculation	Inclusion and exclusion criteria	Randomization	Allocation concealment	Reporting of animals excluded from analysis	Blinded assessment of outcome	Reporting potential conflicts of interest and study funding	Quality score
Hao Y J 2007 [27]	N/A	N/A	N/A	N/A	N/A	N/A	N/A	0
Wei J S 2014 [28]	N/A	N/A	+	N/A	N/A	N/A	+	2
Chen L 2013 [29]	N/A	N/A	N/A	N/A	N/A	+	+	2
Namkung-Matthai H 2001 [30]	+	N/A	N/A	N/A	N/A	N/A	N/A	1
Kuo Y J 2014 [31]	N/A	N/A	+	N/A	N/A	N/A	+	2
Fu L 2009 [6]	N/A	N/A	+	N/A	+	N/A	+	3
Zhang C 2009 [32]	N/A	N/A	+	N/A	N/A	N/A	N/A	1
Zhang G 2014 [33]	N/A	N/A	+	N/A	N/A	N/A	N/A	1
Xiao D M 2007 [34]	N/A	N/A	+	N/A	N/A	N/A	N/A	1
Li Z Q 2009 [35]	N/A	N/A	+	N/A	+	N/A	N/A	2
Jin W J 2013 [36]	N/A	N/A	+	N/A	N/A	N/A	+	2
Huang X H 2012 [37]	N/A	N/A	+	N/A	N/A	N/A	N/A	1
Rong J M 2012 [38]	N/A	N/A	+	N/A	+	N/A	+	3
Zhang C 2008 [39]	N/A	N/A	+	N/A	+	N/A	N/A	2
Dai G C 2013 [40]	N/A	N/A	+	N/A	+	N/A	N/A	2
Wang X P 2014 [41]	N/A	N/A	+	N/A	+	N/A	+	3
Qiu Z J 2010[42]	N/A	N/A	+	N/A	+	N/A	N/A	2
Cao G L 2014 [43]	N/A	N/A	+	N/A	N/A	N/A	N/A	1
Sha M 2009 [44]	N/A	N/A	N/A	N/A	+	N/A	+	2
Chow S K 2014 [45]	N/A	+	+	N/A	N/A	N/A	+	3
Cheung W H 2012 [46]	N/A	N/A	+	N/A	N/A	N/A	+	2
Blokhuis T J 2012 [48]	N/A	N/A	+	N/A	+	N/A	+	3
Mohamad S 2012 [47]	N/A	N/A	+	N/A	N/A	N/A	+	2
Wang L 2005 [49]	N/A	+	+	N/A	+	N/A	N/A	3
Estai M A 2011 [50]	N/A	N/A	+	N/A	N/A	N/A	+	2
Shi H F 2010 [51]	N/A	+	+	N/A	+	N/A	+	4

N/A, not available; +, mentioned.

### Assessment of biomechanical test values

In the data analysis process, we found that the biomechanical data of one study was abnormally higher than that of the other studies at the same time point (20 times higher than the average value at D28 PF and 5 times higher than the average value at D84 PF) [33]. The interquartile range (IQR) method was used to analyze the data. It was found that the data from that study were beyond the range of the confidence intervals; therefore, the study was removed. Data from 25 studies were thus included in the final analysis.

### Fracture healing was delayed and biomechanical property of fractured femur decreased in OVX rats

**Fig 2:**
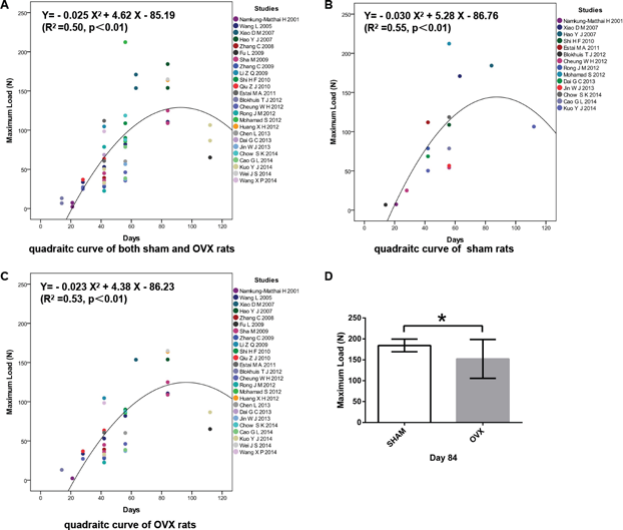
Osteoporotic fracture healing was delayed and biomechanical property decreased in OVX rats. (A) In the fitted parabola of both osteoporotic fractures and sham fractures, the predicted fracture healing time was 92.4 d, and the predicted maximum load was 128.30 N. (B) In the fitted parabola of sham fracture, the predicted maximum load was 145.56 N, and the predicted the fracture healing time was 88.0 d. (C) In the fitted parabola of osteoporotic fracture only, the predicted maximum load was 122.30 N, and the predicted fracture healing time was 95.2 d. X, fracture healing time; Y, maximum load. (d) Maximum load of fractured femurs in OVX rats was significantly lower that of the sham group at D84 PF (p<0.05).

Thirty-six values of the maximum load of OVX rats from 25 studies and 16 values of sham rats from 14 studies at different time points were included to fit the quadratic curve to assess the relationship between the maximum load of the healing femurs and the length of healing time during the process of fracture healing. In the estimated curves, mechanical strength initially gradually increased over fracture healing time and then started to decrease at a certain time point. In the fitted parabola, the abscissa value of the highest point was considered to be the true maximum load of the healed femurs during fracture healing, and the ordinate value of the highest point was considered to be the healing time for functional recovery of the fractured femurs. In the fitted parabola of both osteoporotic fractures and sham fractures, the predicted fracture healing time was 92.4 d, and the predicted maximum load was 128.30 N (Fig 2A). In the fitted parabola of sham fractures, the predicted maximum load was 145.56 N, and the predicted fracture healing time was 88.0 d (Fig 2B). In the fitted parabola of osteoporotic fractures only, the predicted maximum load was 122.30 N, and the predicted fracture healing time was 95.2 d (Fig 2C). The results indicated that the fracture healing process was delayed (S1 Fig) and the biomechanical quality of healed femurs decreased in OVX rats compared with sham rats, which was consistent with the results of pathological studies and clinical observations.

To confirm the difference in biomechanical property of healed bones between OVX rats and sham rats, the maximum loads from all of the studies with OVX rats and sham rats at day D84 PF were chosen and analyzed because D84 PF was the closest time point to the predicted fracture healing time and the biomechanical values of D84 PF had the greatest impact on the direction of the curve. The values of maximum loads from osteoporotic fracture and sham fracture studies were extracted and analyzed. Six studies with OVX rats and one study with sham rats were involved [27, 28, 37, 41, 44, 49]. Two different intramedullary nails were used in one of the six studies, and two sets of biomechanical values were included [44]. It was confirmed that the maximum load of healing femurs in OVX rats was lower than that in sham rats at D84 PF, which was consistent with the results of the fitted quadratic curves (Fig 2D).

### Identification of factors affecting osteoporotic fracture healing in OVX rat models

**Fig 3:**
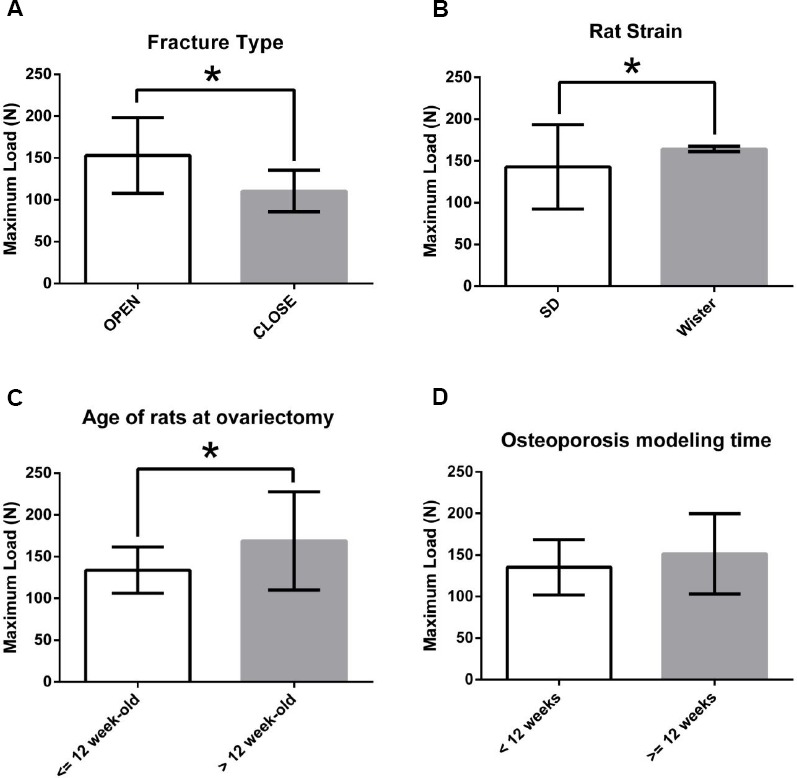
Identification of factors affecting osteoporotic fracture healing in OVX rats at D84 PF. (A) Maximum load of fractured femurs in OVX rats with closed fractures was significantly lower than that of OVX rats with open fractures at D84 PF (p<0.05). (B) Maximum load of fractured femurs in SD rats was significantly different from that of Wister rats at D84 PF (p<0.05). (C) Studies were divided into two groups according to the age of the rats when ovariectomized. Maximum load of OVX rats ovariectomized at < = 12 weeks old was lower than that of OVX rats ovariectomized >12 weeks old at D84 PF (p<0.05). (D) Studies were divided into two groups according to the length of time from ovariectomy to femur fracture surgery, <12 weeks or > = 12 weeks. There was no significant difference in femur maximum load between these two groups at D84 PF (p>0.05).

To further identify the factors affecting osteoporotic fracture healing, the maximum loads of fractured femurs at D84 PF were extracted from all of the studies with rat OVX models and were divided into two groups according to fracture type, rat strain, the age of the rats at ovariectomy and the osteoporosis modeling time. At D84 PF, the average maximum load of the OVX rats with open femur fractures was significantly higher than that of OVX rats with closed femur fractures (Fig 3A). The average maximum load of OVX Wister rats was higher than that of OVX SD rats (Fig 3B). In addition, the studies were divided into two groups according to the age of the rats when ovariectomy was performed, < = 12 weeks old and >12 weeks old. The maximum load of OVX rats who received an ovariectomy at < = 12 weeks old was lower than that of OVX rats who were ovariectomized at >12 weeks old (Fig 3C). The studies were also divided into two groups according to the length of time from the ovariectomy to femur fracture surgery, <12 weeks or > = 12 weeks. There was no significant difference in femur maximum load between these two groups (Fig 3D). To further analyze the impact of fracture type, rat strain and age of the rats at ovariectomy on osteoporotic fracture healing, the quadratic curves between the maximum load and the fracture healing time were also fitted.

### Osteoporotic fracture healing was delayed in OVX rats with closed fractures

**Fig 4:**
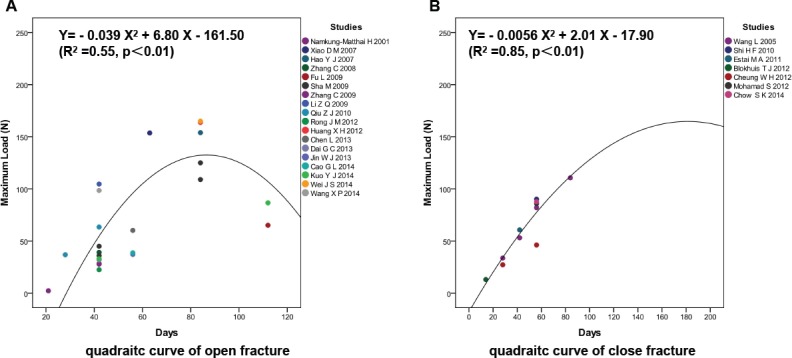
Osteoporotic fracture healing was delayed in OVX rats with closed fractures. (A) In the quadratic curve of open femur fracture, the predicted maximum load was 134.91 N and the predicted fracture healing time was 87.2 d. (B) In the quadratic curve of closed femur fracture, the predicted maximum load was 162.47 N and the predicted fracture healing time was 179.5 d.

Eighteen studies with 25 biomechanical values of OVX rats with open femur fractures and seven studies with 11 biomechanical values of OVX rats with closed femur fractures at different time points were included. In the quadratic curve for open femur fracture (Fig 4A), the predicted maximum load was 134.91 N, and the predicted fracture healing time was 87.2 d. In the quadratic curve of closed femur fracture (Fig 4B), the predicted maximum load was 162.47 N, and the predicted fracture healing time was 179.5 d. Osteoporotic fracture healing was delayed and biomechanical property decreased in OVX rats with closed fractures (S2 Fig).

### Osteoporotic fracture healing was not affected by the strain of the rat, but the biomechanical property differed

**Fig 5:**
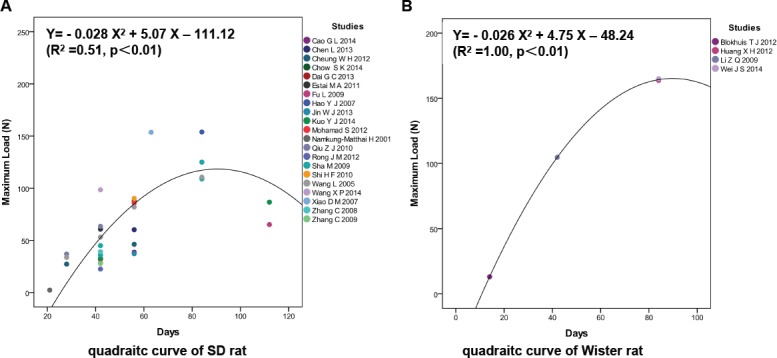
Osteoporotic fracture healing was not affected by the strain of rat. (A) In the quadratic curves of SD rats, the predicted fracture healing time was 90.54 d and the predicted maximum load was 118.39 N. (B) In the quadratic curve of Wister rats, the predicted fracture healing time was 91.35 d and the predicted maximum load was 168.71 N.

Twenty-one studies with 32 biomechanical values of OVX SD rats and four studies with 4 biomechanical values of OVX Wister rats were assessed to identify whether the strain of rat had an effect on osteoporotic fracture healing. In the quadratic curve of SD rats, the predicted fracture healing time was 90.54 d, and the predicted maximum load was 118.39 N (Fig 5A). In the quadratic curve of Wister rats, the predicted fracture healing time was 91.35 d, and the predicted maximum load was 168.71 N (Fig 5B). Although no obvious difference in fracture healing time was shown (S3 Fig), the maximum load of SD rats was significantly lower than that of Wister rats, as seen in the predictions of the quadratic curve and values at D84 PF; this suggests that the Wister rat itself may have better bone strength than SD rats.

### Osteoporotic fracture healing was delayed in rats < = 12 weeks old at ovariectomy

**Fig 6:**
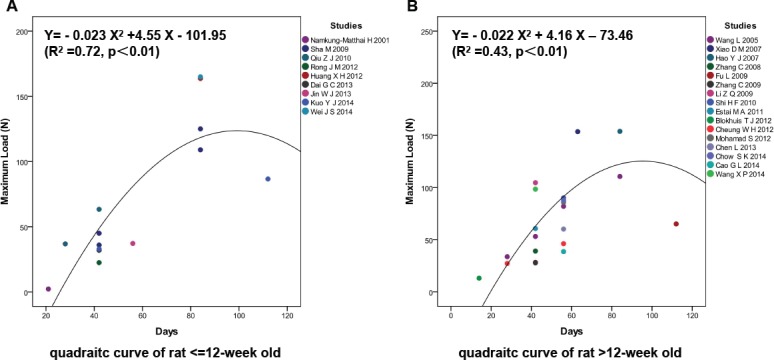
Osteoporotic fracture healing was not delayed in rats < = 12 weeks old at ovariectomy. (A) In the < = 12-week-old group, the predicted fracture healing time was 98.91 d and the predicted maximum load was 123.05 N. (B) In the >12-week-old group, the predicted fracture healing time was 94.55 d and the predicted maximum load was 123.20 N.

The studies with OVX rats were divided into two groups (< = 12 weeks old and >12 weeks old) according to the ages of the rats at the time of ovariectomy. Nine studies with 14 biomechanical values of rats < = 12 weeks old at ovariectomy and 16 studies with 22 biomechanical values at different time points on rats >12 weeks old were included. The quadratic curves of the two groups were fitted. In the < = 12-week-old group (Fig 6A), the predicted fracture healing time was 98.91 d and the predicted maximum load was 123.05 N. In the >12-week-old (Fig 6B) group, the predicted fracture healing time was 94.55 d, and the predicted maximum load was 123.20 N. This suggested that osteoporotic fracture healing could be delayed in the rats receiving an ovariectomy at < = 12 weeks old but that the final bone strength would be approximately similar (S4 Fig).

## Discussion

Bone fracture healing was delayed and callus quality was decreased by osteoporosis [30, 53]. However, the process of osteoporotic fracture healing reflected by biomechanical tests is still unknown, despite the fact that biomechanical tests are more critical in bone quality evaluation. Many studies have performed biomechanical tests according to imaging studies or histomorphological observations, which cannot truly reflect the biomechanical properties of the fractured bone and results in dispersed assessing time points [30, 54]. In this study, the quadratic curve showed that during osteoporotic fracture healing, the maximum load on the fractured femur increased rapidly early on, gradually reached a peak point and then started to decline. This suggests that the bone strength of the fractured bone will reach a peak point at a certain stage of fracture healing. With callus remodeling and development of osteoporosis, the strength of the fractured bone will then slowly decline. The corresponding fracture healing time of the peak strength could be considered the time point of functional recovery of osteoporotic fracture. In addition, the fitted curve also confirmed that osteoporosis not only delayed the process of fracture healing but also decreased the biomechanical property of the fractured bone. Based on these findings, it is suggested that in the assessment of femur fracture healing by biomechanical test in OVX rats, time points at or later than D95 PF should at least be included, and the comparable maximum load of the healed femur in OVX rat should be approximately 122.3 N.

We also identified several factors that may affect osteoporotic fracture healing including fracture type, rat strain, age of the rats at ovariectomy and osteoporosis remodeling time. There were two fracture types reported in the included studies, open fracture and closed fracture. Open fracture is usually established by creating a transverse fracture through surgery, and closed fracture is typically induced with a guillotine device or a three-point bending apparatus resulting in comminuted fracture due to the osteoporosis. One of the most dominant mechanical factors has been reported to be the fracture geometry, described by fracture type and gap size [55]. Comminuted fractures and fractures with large butterfly fragments can delay the fracture healing time, and fracture repair capacity can also be decreased with an increase in fracture gaps [56, 57]. In addition, muscle can make a direct cellular contribution to bone, acting as a "secondary periosteum" when the periosteum is damaged or absent in an open fracture [58]. Myogenic progenitors in muscle make substantial contributions to every stage of the open fracture healing process but not to that of closed fracture healing. The intact periosteum may act as a physical barrier that prevents the invasion of myogenic progenitors into the fracture site [59]. Consistent with these findings, our results showed that osteoporotic fracture healing was significantly delayed in rats with closed fracture compared with rats with open fracture. When open femur fracture is used in OVX rats, the D87 PF time point should be considered for biomechanical tests. The predicted fracture healing time in OVX rats with a closed femur fracture was D179 PF. Nevertheless, because all of the time points in the enrolled studies on closed fracture were before D87 PF and no data after D87 PF could be adopted, the fitted quadratic curve for closed fractures was defective.

The maximum loads of SD rats were lower than that of Wister rats because different rat strains may have different bone mineral density, bone size and biomechanical bone strength [60]. However, the processes of osteoporotic fracture healing of the two rat strains are not significantly different. When SD rats and Wister rats are used to study osteoporotic fracture healing in OVX models, the same time points for mechanical tests can be chosen, but the values of the biomechanical indexes are not comparable between SD rats and Wister rats.

Female rats reach sexual maturity and achieve peak bone mass at the age of 12 weeks and are commonly ovariectomized at this age [61, 62]. There were only three studies in which ovariectomy were performed in rats <12-weeks old. None of them recorded maximum load at D84 PF. Therefore, we divided the included studies into < = 12-week-old group and >12-week-old group. Bone loss is known to be more pronounced in rats who receive an ovariectomy before sexual maturity, and the younger the rat is when ovariectomized, the more serious is the bone loss [63]. Fracture healing was delayed in OVX rats in this study. Therefore, it is suggested that fracture healing may be delayed in the rats which were ovariectomized before sexual maturity. This review found that osteoporotic fracture healing could be delayed in rats who were < = 12 weeks old when ovariectomized but that the maximum loads were approximative. Thus at D84 PF, compared with the group of rats ovariectomized at < = 12 weeks old, the fracture healing of the >12-week-old group was closer to the peak point of maximum load and their bone strength was better.

In many studies, fractures are established in rats 12 weeks after ovariectomy because significant bone loss at different sites can definitively be induced at this time [64–66]. In this review, there was no obvious difference in the maximum loads of fractured femurs in OVX rats who had a remodeling time of <12 weeks compared with those with > = 12 weeks. This suggests that the osteoporosis modeling time may not be the primary factor affecting the biomechanical properties of femur fracture healing in OVX rats. However, obvious bone loss in the proximal femur of rats can be induced as early as 5 weeks after ovariectomy, regardless of the age of the rat, after which the rate of bone loss appears to stabilize at a certain level [67]. In this review, the majority of the analyzed studies performed the femur fractures more than 6 weeks after the ovariectomy. Thus when the maximum loads between the <12 weeks and > = 12 weeks groups at D84 PF were compared, no obvious differences were observed. Therefore, more studies on OVX rats with an osteoporosis modeling time of less than 5 weeks are needed to clarify the true effects of osteoporosis modeling time on osteoporotic fracture healing.

There are also other factors that may lead to variance in biomechanical test, such as the type of biomechanical test, loading speed and the separation between two bottom supports. These experimental settings differed in the included studies, and even some studies didn’t mention these details. For outcome parameters, besides maximum load, only a few studies recorded stiffness, elastic modulus and stress. Overall, these limitations in the study design and performance led to difficulties in a comprehensive analysis of factors affecting biomechanical test.

## Conclusions

In summary, we systematically reviewed studies that used biomechanical tests to assess osteoporotic femur fracture healing with OVX rat models. Our results showed that fracture healing was delayed and biomechanical property of fractured bone decreased by osteoporosis. It is suggested that in the assessment of femur fracture healing by biomechanical test in OVX rats, D95 PF should be included as one of the time points. Second, compared with open fracture, our findings showed that the osteoporotic femur fracture healing in OVX rats with closed fractures was delayed. Third, although there may be differences in bone strength between different strains of rats, the process of osteoporotic fracture healing was not affected by the strain of the rat. Finally, more studies are still needed to clarify the impact of the age of the rat at ovariectomy and of the osteoporosis modeling time on osteoporotic fracture healing.

## Supporting Information

S1 PRISMA Checklist (DOC)

S1 Fig The combined quadratic curves between maximum load and fracture healing time of sham rats and OVX rats. (TIF)

S2 Fig The combined quadratic curves between maximum load and fracture healing time of OVX rats with open fractures and closed fractures. (TIF)

S3 Fig The combined quadratic curves between maximum load and fracture healing time of SD rats and Wister rats. (TIF)

S4 Fig The combined quadratic curves between maximum load and fracture healing time of rats < = 12 weeks old and >12 weeks old at the time of ovariectomy. (TIF)
