# Supplementary figures and images for: Biomechanical Characteristics of Osteoporotic Fracture Healing in Ovariectomized Rats: A Systematic Review
Source: PLoS One. 2016 Apr 7;11(4):e0153120. doi: 10.1371/journal.pone.0153120 (PMC4824477; doi:10.1371/journal.pone.0153120)

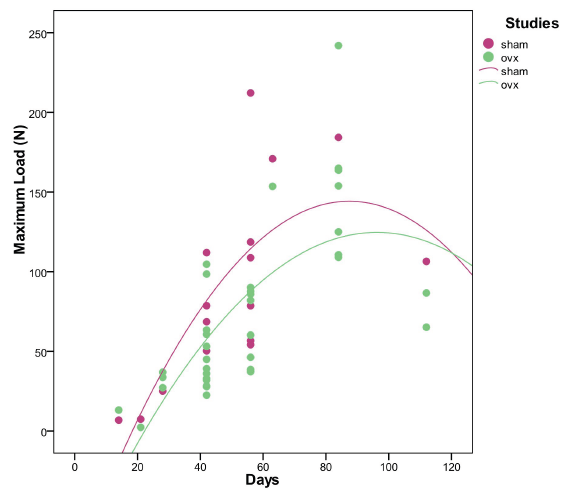

Supplement: S1 Fig — (TIF) [file pone.0153120.s002.tif]

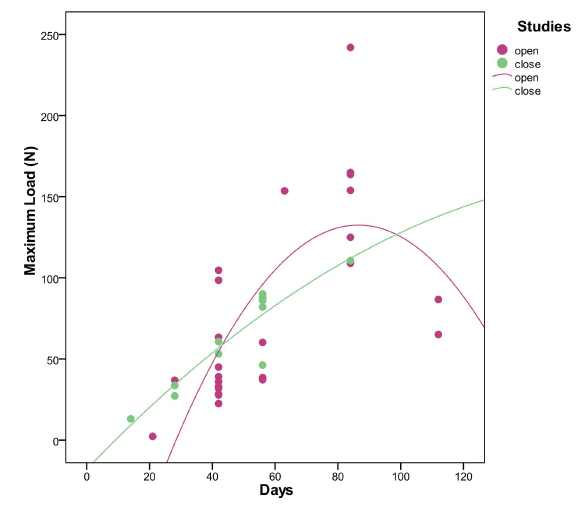

Supplement: S2 Fig — (TIF) [file pone.0153120.s003.tif]

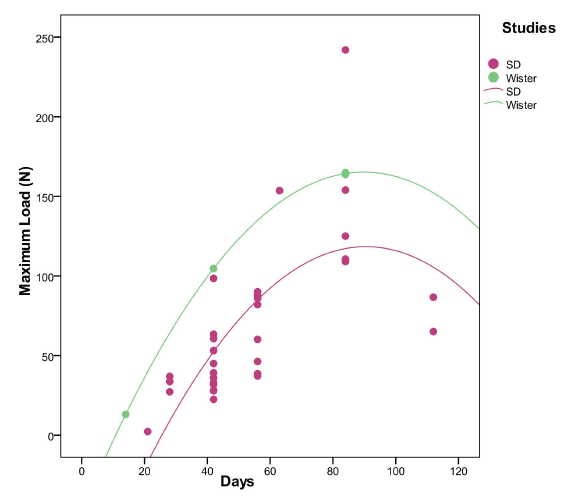

Supplement: S3 Fig — (TIF) [file pone.0153120.s004.tif]

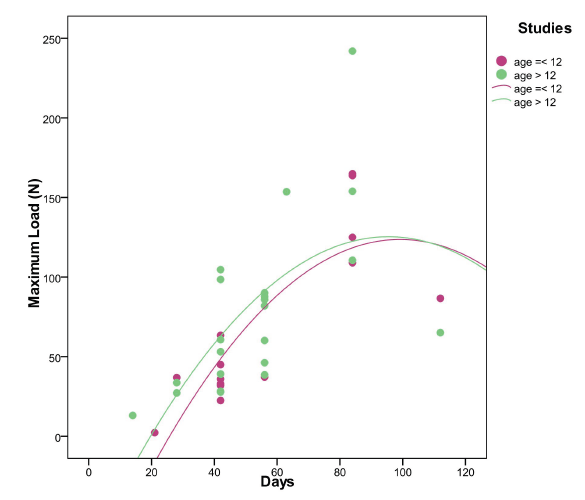

Supplement: S4 Fig — (TIF) [file pone.0153120.s005.tif]
